# Diet-Related Risk Factors for Leprosy: A Case-Control Study

**DOI:** 10.1371/journal.pntd.0003766

**Published:** 2015-05-12

**Authors:** Inge Wagenaar, Lisanne van Muiden, Khorshed Alam, Robert Bowers, Md. Anwar Hossain, Kolpona Kispotta, Jan Hendrik Richardus

**Affiliations:** 1 Department of Public Health, Erasmus Medical Center, Rotterdam, The Netherlands; 2 Wageningen University and Research Centre, Wageningen, The Netherlands; 3 Rural Health Program, The Leprosy Mission International-Bangladesh, Nilphamari, Bangladesh; 4 Nilphamari Training Center, The Leprosy Mission International-Bangladesh, Nilphamari, Bangladesh; University of Texas Medical Branch, UNITED STATES

## Abstract

**Background:**

Food shortage was associated with leprosy in two recent studies investigating the relation between socioeconomic factors and leprosy. Inadequate intake of nutrients due to food shortage may affect the immune system and influence the progression of infection to clinical leprosy. We aimed to identify possible differences in dietary intake between recently diagnosed leprosy patients and control subjects.

**Methods:**

In a leprosy endemic area of Bangladesh, newly diagnosed leprosy patients and control subjects were interviewed about their socioeconomic situation, health and diet. Dietary intakes were recorded with a 24-hour recall, from which a Dietary Diversity Score (DDS) was calculated. Body Mass Index (BMI) was calculated and Household Food Insecurity Access Scale (HFIAS) was filled out for every participant. Using logistic regression, a univariate, block wise multivariate, and an integrated analysis were carried out.

**Results:**

52 leprosy cases and 100 control subjects were included. Food shortage was more common, dietary diversity was lower and household food insecurity was higher in the patient group. Patients consumed significantly less items from the DDS food groups ‘Meat and fish’ and ‘Other fruits and vegetables.’ Lower food expenditure per capita, lower BMI, lower DDS and absence of household food stocks are the main factors associated with an increased risk of having leprosy.

**Conclusion:**

Low income families have only little money to spend on food and consequently have a low intake of highly nutritious non-rice foods such as meat, fish, milk, eggs, fruits and vegetables. Development of clinical leprosy could be explained by deficiencies of the nutrients that these foods normally provide.

## Author Summary

Even though leprosy is one of the oldest diseases known to mankind, there is still a lot unknown about its transmission and why some people develop the disease and others do not. Leprosy is often seen as a disease of the poor, but which aspects of poverty are associated with leprosy are still under study. Recently though, food shortage has been identified as a risk factor for leprosy in two socioeconomic studies. In our study, lead in a poor area of Bangladesh, we further investigated this link by interviewing recently diagnosed leprosy patients during a food shortage period. We found that compared to a control population, leprosy patients have less money to spend on food, have less household food stocks and have a less diverse diet. The patient group had a lower consumption of highly nutritious foods such as meat, fish, eggs, milk, fruits and vegetables. An inadequate diet for a longer period of time leads to nutrient deficiencies. The body’s immune system, however, needs proteins, vitamins and minerals to effectively fight off infections. We conclude that people who are living in poverty and who are not able to get an adequate, diverse diet have a higher chance of developing leprosy.

## Introduction

Leprosy, an infectious disease caused by *Mycobacterium leprae*, affects skin and nerves and can lead to deformities of the hands, feet and face. The disease remains a public health problem in underdeveloped areas in the world, and is therefore known as a disease of the poor. Our understanding of risk factors for the transmission of *M*. *leprae* and the development of leprosy disease is not complete. One of the reasons is the long incubation period, which is on average 2–5 years. It is therefore difficult to investigate causal relationships between circumstances at the time of infection and the onset of clinical symptoms years later. The most important known determinant for contracting leprosy is being a household contact of a leprosy patient, which carries a five to eight times higher risk of contracting leprosy [1,2]. The specific risk factors that determine the risk for contacts include the Ridley-Jopling leprosy classification of the index patient, physical distance to the patient and age of the contact [3]. However, in endemic regions the majority of new leprosy patients are not close contacts of a known leprosy case [4,5]. Another possible risk factor is poverty, although not all poor countries have high leprosy prevalence rates. It is even the case that Brazil, an economically emerging country, has one of the highest new case detection rates in the world [6]. It remains unclear which aspects of poverty are associated with leprosy susceptibility and the progression to clinically detectable disease. Two recent case-control studies investigating socioeconomic factors in relation to leprosy found that food shortage was associated with leprosy. The setting of one of these studies was a poor, high leprosy endemic area in Brazil [7]. Among other factors, having experienced food shortage at any time in life was related to leprosy. The other study was set in two leprosy endemic districts in Bangladesh. In this study, food shortage in the past year was the only factor significantly associated with the clinical manifestation of leprosy [8].

The definition of food shortage used in the Bangladesh study was: ‘the period in which a family had to reduce the number of meals a day, or reduce the intake of foods other than rice’. This is most likely to occur when rice prices are high, household food stocks depleted and income is low due to lack of labor opportunities. Multiple studies in Bangladesh documented that this situation typically arises in the period between September and the end of November, just before the major harvest Aman in December [9–12].

Food shortage worsens the often already inadequate intake of micro- and macronutrients. Nutritional deficiencies impair the immune system and thus the defense of the body against infections [13,14]. The risk of contracting subclinical *M*. *leprae* infection is not necessarily increased by food shortage, but it could facilitate the progression from infection to the clinical presentation of leprosy.

The findings of the two above-mentioned studies raise many questions regarding the exact mechanisms behind the relationship between food shortage and leprosy. Literature on this subject is scarce, and to further examine this relationship we designed a case-control study in rural Bangladesh during an expected period of food shortage. The aim was to assess possible differences in dietary intake between recently diagnosed leprosy patients and control subjects without the disease that could lead to hypotheses on immunological mechanisms underlying the clinical development of leprosy.

## Methods

### Study population and sampling

This case-control study was conducted in October and November 2013 in northwest Bangladesh, in the mainly rural and agricultural Nilphamari and Rangpur districts. These are among the poorest regions in Bangladesh [15], and leprosy is still endemic in this area. In 2012, 512 new leprosy cases were found in these districts, which have a total population of four million (data from the Rural Health Program, Nilphamari).

Data of all patients diagnosed with leprosy in the first half of 2013 were gathered from the Rural Health Program database in Nilphamari, which is run by The Leprosy Mission International, Bangladesh (TLMIB). Our aim was to include an equal number of men and women, to have an even age distribution and take in only one person (case or control) per household. Furthermore, we only included patients aged between 18 and 50 years and with help of field staff we pre-selected patients with a low risk of stigma possibly caused by a home visit. Of the 180 patients, 92 were eligible for this study, 64 were outside the age bracket criterion and 24 were excluded to avoid the risk of stigma of a home visit. Controls were selected from a random cluster sample of the population, originally composed for the COLEP study [16]. Out of the 13 sub-districts in the area, 20 clusters were formed, each containing 1000 randomly selected people [17]. We selected three clusters that could be reached within approximately one hour by motorcycle from the TLMIB Leprosy Center in Nilphamari; two clusters in Nilphamari district, of which one rural and one suburban, and one rural cluster in Rangpur district. From each cluster, 34 controls were randomly selected using a computerized sampling method, with an even distribution of men and women. Controls were excluded if they or a household member had ever been diagnosed with leprosy and if they were under 18 or over 50 years of age. When a control subject was not available at the time of our first visit, we made two more attempts. If the control subject was still not available the third time, a neighbor of similar age was invited to participate.

### Ethics statement

Ethical approval for the study protocol was given by the institutional review board of TLMI Bangladesh, Nilphamari. Informed written consent was obtained from all participants.

### Data collection

Data on patients and controls were collected by means of a structured questionnaire, a 24-hour dietary recall and anthropometric measures. The questionnaires for cases and controls were developed in English, translated separately by two translators to Bengali and each of them translated their colleague’s version back to English. The translations were discussed and the questionnaire was optimized. The questionnaire was pre-tested on patients and controls and adjusted where necessary. The questions of the Household Food Insecurity Access Scale (HFIAS) were kindly provided in Bengali by the International Centre for Diarrheal Disease Research, Bangladesh (ICDDR,B). Data were collected during household visits by two staff members of the TLMIB Nilphamari Training Center, both were trained field workers fluent in Bengali and English.

The questionnaire focused on demographic, socioeconomic and health data of the subjects and their households. The questions dealt with the occupation of the income generator, household size (defined as the number of people eating in the house), average income and income variation, self-classification on a poverty scale (very poor to rich), land ownership, food expenditure, any health problems other than leprosy in the past year and the presence of a BCG scar. If a patient’s income had changed since the diagnosis of leprosy, the pre-diagnosis income was used in the analyses. Income and food expenditure were then calculated per capita. Second, the HFIAS was administered [18]. This validated tool monitors problems with food access, dietary modifications and concerns about food insecurity over the past four weeks. Finally, subjects were asked questions about their history of food shortage, their household food stocks, and details of their coping mechanisms such as reducing the number or variety of meals. For the sake of comparability, these questions and the definition of food shortage were based on the study of Feenstra et al. [8]. Dietary intakes were assessed by a 24-hour recall, following the Food and Agriculture Organization (FAO) guidelines for measuring individual dietary diversity [19]. Subjects were asked to list the foods consumed during the previous day, starting from waking up in the morning. Details of all meals and snacks, consumed inside and outside the house during the full day, were recorded in chronological order. To be as accurate as possible, subjects were prompted about drinks, snacks and food consumed in and outside the house. Recipes of mixed meals were obtained to ascertain that all ingredients were recorded. The 24-hour recalls were carried out on weekdays, with exception of atypical holidays. Therefore, no interviews were held in the week after Eid al-Adha (Festival of Sacrifice). Also, two focus group discussions were held with women to gather information on commonly used ingredients and preparation methods.

Weight of subjects was measured using a portable scale and assessed to the nearest 0.5 kg. Subjects were asked to remove shoes. Height was measured with a measuring tape while the subject was standing barefooted with his/her back straight against a wall. Body mass index (BMI) was calculated as weight (kg)/ height^2^ (m). Subjects were identified as underweight when their BMI was lower than 18.5 kg/m^2^. From the 24-hour recalls, the Dietary Diversity Score (DDS) was calculated [19]. The DDS is a simple count of the food groups consumed by the subject, and is increasingly used to measure dietary quality [20,21]. Nine food groups were included in this study: ‘Starchy staples’, ‘Dark green leafy vegetables’, ‘Other vitamin A rich fruits and vegetables’, ‘Other fruits and vegetables’, ‘Organ meat’, ‘Meat and fish’, ‘Eggs’, ‘Legumes, nuts and seeds’ and ‘Milk and milk products’. Vitamin A rich fruits and vegetables were defined as containing a minimum of 60 RAE/100 g (RAE stands for retinol activity equivalents) [19]. The condiments garlic and chilies were not counted, because the amount consumed per person was likely to be very low. The possible score ranged from 0 to 9. A score ≥5 was considered as adequately diverse [22].

### Analysis

Before statistical analysis, a framework was built using four blocks comprised of several related variables: Demographic factors (age, sex, religion, district, and household size); socioeconomic factors (income, food expenditure, poverty classification, occupation, and land ownership); health factors (diseased in the last year, BCG scar, and BMI); and diet-related factors (HFIAS, DDS, food shortage in the past year, food shortage at any time in life, and household food stocks).

Statistical analyses were performed using SPSS (version 22, SPSS Inc., Chicago, IL). All analyses were done using logistic regression, with case/control as dependent variable. Income and food expenditure were log transformed to normalize their distribution. To reduce the effect of matching, age and sex were continuously adjusted for in the univariate, multivariate per block and integrated analyses. Univariate analysis was carried out first, and the variables significantly (p<0.10) associated with leprosy were included in a multivariate backward stepwise logistic regression per block. The variables that remained statistically significant (p ≤0.05) in these multivariate analyses were considered as the main result. Finally, for the integrated analysis, the significant variables of each block (p<0.10 in the Wald Chi Square test) were combined and again backward stepwise logistic regression was carried out, this time using a p-value of <0.05 as statistically significantly contributing to the model.

## Results

Fifty-two leprosy cases and 100 control subjects were interviewed during home visits. The majority of the leprosy patients had paucibacillary (PB) leprosy (65%), was released from multidrug treatment (56%) and had no leprosy-related disabilities (71%). Seventy-one percent of the controls were acquainted with at least one person diagnosed with leprosy, in most cases a neighbor (59%). Other demographic and socioeconomic characteristics of the patient and control groups are shown in Table 1. In general, the socioeconomic variables for the patient group were less favorable.

**Table 1:** Demographic, socioeconomic and health characteristics.

		Cases (n = 52)	Controls (n = 100)
Sex	Male	29(56%)	48(48%)
Age	Mean (Years)	35.0±9.5	33.3±10.4
	15–29	25.0%	37.0%
	30–44	32.7%	27.0%
	45–60	42.3%	36.0%
District	Nilphamari	27(51.9%)	67(67.0%)
	Rangpur	25(48.1%)	33(33.0%)
Household size	Mean	4.6±1.4	5.2±2.1
Income	Household mean (BDT)	5115±3621	8177±6398
	Per capita mean (BDT)	1180±886	1766±2011
Income variation	Mean (BDT)	3827±2852	5234±5719
Food expenditure	Household mean (BDT)	4545±2323	6540±3435
	Per capita mean (BDT)	1046±530	1340±841
Land owned	Landowner	8(15.4%)	34(34.0%)
	Mean size (m^2^)	387±1214	3161±6820
BMI^1^	Mean (kg/m^2^)	20.3±3.1	21.6±3.0
	Underweight	25.0%	14.0%
	Normal weight	67.3%	72.0%
	Overweight	7.7%	14.0%
Leprosy	Paucibacillary	34(65.4%)	-
	Multibacillary	18(34.6%)	-
Disability grade	0	37(71.2%)	-
	1	9(17.3%)	-
	2	6(11.5%)	-

^1^ Body Mass Index, categories: underweight <18.5, normal: 18.5–25, overweight: >25; BDT: Bangladesh Thaka, 100 BDT = ± $1.28

**Fig 1:**
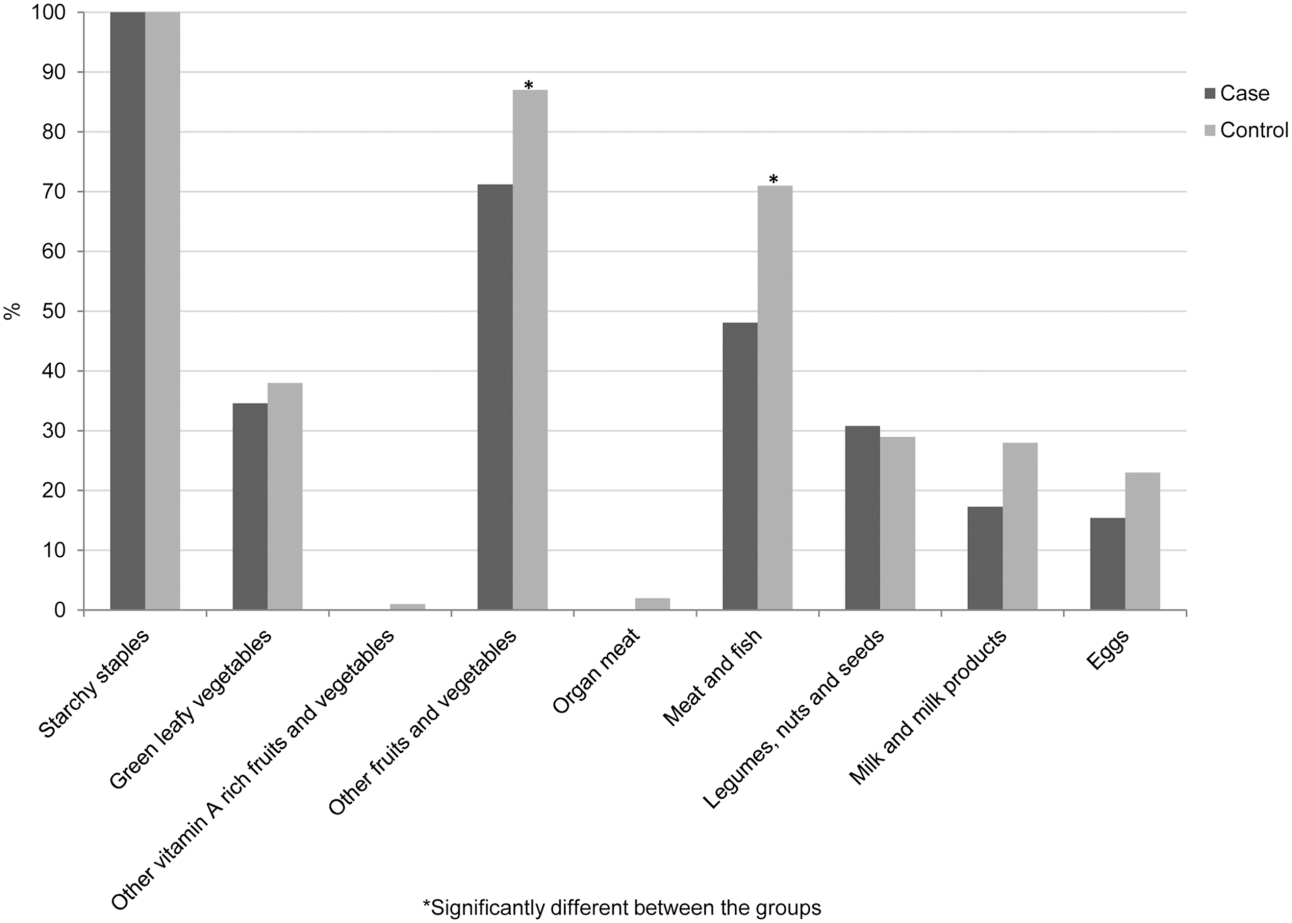
Proportions of leprosy patients and controls consuming items from the 9 food groups.

**Fig 2:**
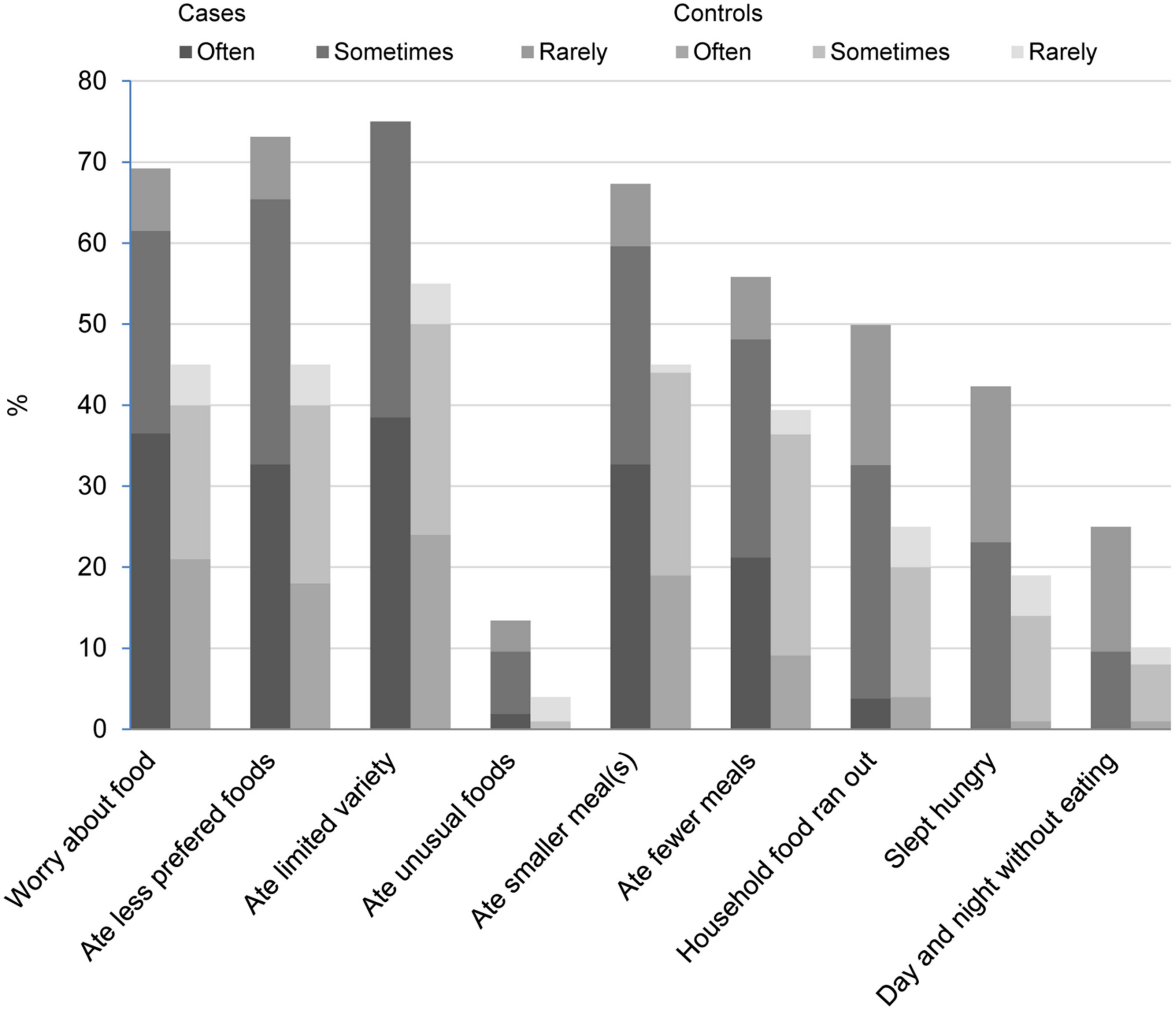
Frequency-of-occurrence for each Household Food Insecurity Access Scale item for leprosy patients and controls.

Table 2 provides detailed data on food shortage, food security and coping mechanisms, from which it becomes evident that leprosy patients are once more in a disadvantaged position. The average DDS was significantly lower in the patient group, who consumed significantly less items from the groups ‘Meat and fish’ and ‘Other fruits and vegetables’ than the control group did (Fig 1). Control subjects consumed more items from the groups ‘Milk and milk products’ and ‘Eggs’ as well, but in this regard there was no significant difference with the patient group. Adequate dietary diversity (DDS ≥5) was reached by 25% of the control subjects and 17.3% of the patients. Food insecurity, measured by HFIAS, was more severe in the patient group, with a mean score of 10.2 and 6.4 for patient and control group, respectively (p = 0.003). Fig 2 gives the proportions and the severity of food insecurity for each item of the scale for both groups. Food stocks were present in 74% of the controls’ households, lasting on average for 32 days, and in 51% of the patients’ households, sufficient for 15 days on average.

**Table 2:** Details on food shortage, coping mechanisms and household food stocks.

		Cases (n = 52)	Controls (n = 100)
DDS^1^		3.2±1.1	3.8±1.4
Experienced food shortage at any time in life		96.2%	84.0%
Experienced food shortage in past year		80.8%	64.0%
Average duration of food shortage (days)		106±118	99±124
Experienced food shortage in			
March/April		5.7%	5.4%
September/October		54.3%	57.1%
Both periods		40.0%	37.5%
Coping mechanisms			
Reduced variety		16.7%	29.7%
Reduced number of meals		-	3.1
Reduced both variety and number of meals		83.3%	67.2%
Changes in consumption of food items			
Fish	No change	9.5%	1.6%
	Reduced	38.1%	64.5%
	Eliminated	52.4%	33.9%
Meat	No change	7.1%	-
	Reduced	28.6%	51.6%
	Eliminated	64.3%	48.4%
Vegetables	No change	57.1%	75.8%
	Reduced	38.1%	22.6%
	Eliminated	4.8%	1.6%
Fruits	No change	50.0%	53.2%
	Reduced	19.0%	25.8%
	Eliminated	31.0%	21.0%
Lentils	No change	73.8%	72.6%
	Reduced	14.3%	24.2%
	Eliminated	11.9%	3.2%
Egg	No change	59.5%	51.6%
	Reduced	19.1%	35.5%
	Eliminated	21.4%	12.9%
Milk	No change	66.7%	53.2%
	Reduced	14.3%	32.3%
	Eliminated	19.0%	14.5%
HFIAS^2^		10.2±7.4	6.4±7.0
Food secure		19.2%	33.0%
Mildly food insecure		3.8%	9.0%
Moderately food insecure		25.0%	31.0%
Severely food insecure		51.9%	27.0%
Household food stock present		51.9%	74.0%
Mean duration food stock (days)		14.9±34.4	32.0±56.1

^1^ Dietary Diversity Score

^2^ Household Food Insecurity Access Scale, categories according to Coates et al. (2007)

Food shortage, at any time in life as well as in the past year, was reported by many people in both groups, but significantly more often by patients (p = 0.03). Food shortage was most common in the months of September to November leading up to the major harvest in December, Aman. Income was reported to be the lowest of the year during these months by 79% of the subjects. Forty-one percent of interviewees who experienced food shortage in September-October last year reported food shortage as well in the period March-April, before the rice harvest in May/June, Boro. Food shortage in the past year lasted a mean of 101 days, during which one consumed less of certain food products or eliminated these from the diet, and often took fewer meals per day. Note that while control subjects mainly reduced their intake of most foods, patients had to eliminate foods more often. Intake of fish and meat was affected most frequently. People who skipped meals to cope with food shortage took two meals (59%) or sometimes even one meal a day (41%) instead of three meals normally in most cases, with no apparent differences between the patient and control groups.

Regardless of being a case or control, people who had had to cope with food shortage in the past year had a DDS of 3.14 (±1.1) versus 4.59 (±1.3) in people without food shortage (p<0.001). Food insecurity was significantly higher among the people suffering from food shortage: their HFIAS score was on average 10.8, versus 0.6 for people not experiencing food shortage (p<0.001).

The results of the univariate and multivariate analysis are summarized in Table 3, and are addressed below per block.

**Table 3:** Results of univariate logistic regression and multivariate logistic regression per block.

Factors		Cases	Controls	Univariate*			Multivariate		
		n = 52	n = 100	OR	(95% CI)	p-value	OR	(95% CI)	p-value
**Block 1: Demographic factors**									
Age (years)		35.0 ± 9.5	33.3 ± 10.4	1.02	(0.99–1.05)	0.269			
Sex	Male	29 (56%)	48 (48%)						
	Female	23 (44%)	52 (52%)	0.69	(0.35–1.37)	0.294			
Religion	Muslim	40 (77%)	88 (88%)						
	Hindu	12 (23%)	12 (12%)	2.21	(0.90–5.38)	0.082	2.23	(0.92–5.46)	0.079
Household size		4.6 ± 1.4	5.2 ± 2.1	0.83	(0.67–1.02)	0.075	0.82	(0.66–1.02	0.073
**Block 2: Socioeconomic factors**									
Income per capita (log)		2.96 ± 0.27	3.12 ± 0.30	0.10	(0.03–0.44)	0.002			
Food expenditure per capita (log)		2.98 ± 0.17	3.08 ± 0.18	0.02	(0.00–0.22)	0.001	0.03	(0.00–0.36)	0.006
Self-classification						0.005			
	Very poor	17 (33%)	14 (14%)	1.00					
	Poor	21 (40%)	29 (29%)	0.61	(0.24–1.50)				
	Low/middle	11 (21%)	35 (35%)	0.26	(0.10–0.69)				
	Middle	3 (6%)	22 (22%)	0.11	(0.03–0.47)				
	Rich	0 (0%)	0 (0%)	--					
Occupation						0.025			0.058
	Laborer	26 (50%)	28 (28%)	1.00			1.00		
	Shopkeeper	10 (19%)	13 (13%)	0.84	(0.31–2.27)		1.28	(0.44–3.80)	
	Other	8 (15%)	25 (25%)	0.32	(0.12–0.86)		0.44	(0.16–1.22)	
	Farmer	5 (10%)	19 (19%)	0.28	(0.09–0.86)		0.24	(0.07–0.83)	
	Business	3 (6%)	15 (15%)	0.19	(0.05–0.76)		0.31	(0.07–1.34)	
Land						0.042			
	Landless	41 (79%)	58 (58%)	1.00					
	Land leaser	3 (6%)	8 (8%)	0.49	(0.12–1.99)				
	Landowner	8 (15%)	34 (34%)	0.34	(0.14–0.81)				
**Block 3: Health factors**									
Disease other than leprosy	No	24 (46%)	49 (49%)						
	Yes	28 (54%)	51 (51%)	1.12	(0.57–2.22)	0.742			
BCG^1^ vaccination	No	26 (50%)	46 (46%)						
	Yes	26 (50%)	54 (54%)	0.89	(0.45–1.76)	0.743			
BMI^2^ (kg/m^2^)		20.3 ± 3.1	21.6 ± 3.0	0.87	(0.77–0.98)	0.020	0.87	(0.77–0.98)	0.020
**Block 4: Diet-related factors**									
HFIAS^3^ (score 0–27)		10.2 ± 7.4	6.4 ± 7.0	1.08	(1.03–1.13)	0.003			
DDS^4^ (score 0–9)		3.2 ± 1.1	3.8 ± 1.4	0.67	(0.50–0.89)	0.007	0.71	(0.52–0.96)	0.024
Recent food shortage	No	10 (19%)	36 (36%)						
	Yes	42 (81%)	64 (64%)	2.42	(1.07–5.47)	0.034			
Ever food shortage	No	2 (4%)	16 (16%)						
	Yes	50 (96%)	84 (84%)	4.30	(0.93–19.77)	0.061			
Household food stocks	No	25 (48%)	26 (26%)						
	Yes	27 (52%)	74 (74%)	0.38	(0.19–0.78)	0.008	0.45	(0.22–0.95)	0.036

* Adjusted for age and sex;

^1^ Bacillus Calmette-Guérin;

^2^ Body Mass Index;

^3^ Household Food Insecurity Access Scale;

^4^ Dietary Diversity Score

### Block 1: Demographic factors

Both religion and household size were significantly related to leprosy in the demographic block (p<0.10). Hindus were two times more likely to be in the patient group. A larger household was associated with a lower risk on leprosy.

### Block 2: Socioeconomic factors

All variables in this block showed a significant association with leprosy in the univariate analysis. A higher income, land ownership, working as a farmer or a businessman and being part of a low/middle or middle income classified household lead to a decreased risk of leprosy. In the multivariate analysis of this socioeconomic block, step by step income per capita, land ownership, and self-classification were removed. The factors that remained significant were food expenditure per capita (p<0.05) and occupation (p<0.10).

### Block 3: Health factors

BCG vaccination coverage was almost similar in both groups, and therefore did not show a relation to leprosy. BMI was the only significant factor in the uni- and multivariate analysis (p<0.05); a lower BMI increased the risk of leprosy.

### Block 4: Diet-related factors

In the univariate analysis, higher HFIAS, food shortage experienced in the past year and at any time in life were significantly associated with an increased risk of leprosy, while higher DDS and household food stocks reduced the chance of having leprosy. In the multivariate analysis of this block, only DDS and household food stocks remained significant (p<0.05).

### Integrated analysis

In addition to the analyses per block, we considered relationships between the blocks. Therefore, all significant factors from each of the four blocks were analyzed together in a final integrated analysis, presented in Table 4. After stepwise elimination of the least significant factors, two factors remained significantly associated with leprosy: food expenditure (log) and household size (p ≤0.05).

**Table 4:** Results of the integrated logistic regression analyses containing the significant variables of the multivariate analysis per block.

Factors		*	Before backward elimination			*	After backward elimination		
		*	OR*	(95% CI)	p-value	*	OR*	(95% CI)	p-value
Age		*	1.00	(0.97–1.05)	0.797	*	1.02	(0.98–1.05)	0.424
Sex	Male	*	1.00			*	1.00		
	Female	*	0.45	(0.20–1.00)	0.050	*	0.52	(0.25–1.10)	0.086
Religion	Muslim	*	1.00			*			
	Hindu	*	1.41	(0.52–3.88)	0.502	*			
Household size			0.76	(0.55–1.04)	0.084		0.68	(0.52–.89)	0.005
Food expenditure		*	0.02	(0.00–0.45)	0.014	*	0.005	(0.00–.08)	<0.001
Occupation		*			0.294	*			
	Laborer								
	Shopkeeper		2.08	(0.62–6.98)					
	Other		0.59	(0.20–1.72)					
	Farmer		0.47	(0.12–1.89)					
	Business		0.66	(0.13–3.25)					
BMI^1^		*	0.90	(0.78–1.04)	0.163	*			
DDS^2^		*	0.83	(0.58–1.18)	0.299	*			
Household food stocks	No	*	1.00			*			
	Yes	*	0.66	(0.29–1.50)	0.320	*			

*Calculated OR’s are adjusted for all other variables in the column;

^1^ Body Mass Index;

^2^ Dietary Diversity Score

## Discussion

In this study, we set out to identify factors through which food shortage may have an effect on the development of leprosy, and in particular addressed the question whether recently diagnosed leprosy patients and controls had different dietary intakes. Our findings show that leprosy patients have a less favorable position with regard to socioeconomic, health and nutritional factors than a control population. Lower food expenditure per capita, lower BMI, lower dietary diversity score (DDS) and absence of household food stocks were the main factors associated with an increased risk of having leprosy.

Food shortage experienced in the past year was significantly associated with leprosy in the univariate analysis, but not in the multivariate analysis. In our study, the proportions of patients and control subjects experiencing food shortage in the past year were higher than in the study of Feenstra et al.: 80.8% vs. 47.8% for patients and 64.0% vs. 35.7% for the controls, respectively. This is remarkable because the studies were carried out in the same geographical area, used the same definition of food shortage, and no important changes were seen in income and food availability over the four years [23]. In the study of Feenstra et al., food shortage was only one item of an extensive socioeconomic questionnaire, however, while the focus of our questionnaire was entirely on diet and food insecurity. This could possibly explain the higher percentages observed in our study.

### Dietary Diversity Score

Traditionally, a Bengali diet consists mainly of rice. Previous studies found that people from Bangladesh get between 74% and 86% of their energy intake from rice [12,22,24]. This suggests that consumption of nutritious non-rice foods is relatively low, which may explain the low DDSs found in our study. These studies demonstrated that the amount of rice a person consumes remains stable during the year, independent of rice price and season [12,25]. As a result, in the period September-November, when rice prices are high and income is low, expenditures on highly nutritious, generally more expensive, food products are likely to be lower. Consequently, dietary diversity scores are lower during these periods. To our knowledge, this is the first published study investigating the DDS in leprosy patients, and therefore there is no data to compare our results with. However, dietary diversity studies carried out among Bangladeshi women found a mean DDS of 3.6 ±1.1 and 3.4 ±1.1, which are very close to our findings of 3.2 ±1.1 and 3.8 ±1.4 for patient and controls, respectively [22,26].

For the DDS, the condiments chilies and garlic were not counted, in spite of the fact that they are used in almost all dishes according to the women participating in our focus group discussions. Still, the amounts consumed per person were likely to be very low, and therefore the contribution to the diet and dietary diversity should be negligible [22].

### Food expenditure

Food expenditure was another important factor associated with leprosy. However, per capita food expenditure and per capita income were highly correlated (Spearman’s correlation coefficient: 0.81, p<0.001) and additional analyses demonstrated that income and food expenditure are interchangeable in the block- and integrated analysis. The association with leprosy could therefore be just through poverty in general, which is a risk factor often associated with leprosy [27]. Two studies in Bangladesh have linked food expenditure and income to dietary diversity, a variable that was statistically significantly related to leprosy in our study [24,28]. In the study of Thorne-Lyman et al., household dietary diversity increased with increasing food expenditure, and primarily the intakes of animal source foods and fruits increased strongly with higher food expenditures. Our control population had significantly higher food expenditures, and accordingly had higher intakes of these food groups.

### Household size

The second variable that remained statistically significant in the integrated analysis was household size. A larger household size gives a lower risk on leprosy. In the study of Feenstra et al., mean household size was larger in the control group as well, although this was not statistically significant. In theory, however, a larger household could increase the risk of leprosy, since it increases the chance of transmission. Nevertheless, in an Indonesia-based study an increased risk was found only for households larger than seven people [29], while in our study no more than 9 of 152 households counted more than seven people; two (3.8%) in the patient group and seven (7%) in the control group.

### Effect of nutrition on leprosy

The exact role of malnutrition on susceptibility to leprosy and the development to a clinical stage remains unclear [30,31]. A recent review on micronutrients and the immune response in leprosy emphasizes this knowledge gap, as only few studies in this field have been carried out and most of the evidence is derived from other diseases, mainly tuberculosis. Apart from this, previous studies are based either on blood analyses, or focused on diets of (cured) leprosy patients after they developed clinical leprosy. In both cases, it is difficult to determine if leprosy is a cause or a consequence of nutritional deficiencies [32–36]. *Mycobacterium leprae* is an intracellular micro-organism, thus a cell-mediated immune response is important in the defense of the human host [37]. Protein-energy malnutrition, as well as inadequate intake of vitamins and/or minerals are linked to a reduced cell-mediated immunity [38]. With lower intakes of several food groups during food shortage periods, deficiencies may have put leprosy patients in our study at risk for a reduced cell-mediated immunity. Another interesting theory that could possibly apply to leprosy is that of immune reconstitution, a well-known phenomenon in HIV after anti-retroviral therapy [39,40]. When the immune response is restored after a period of suppression, the immune system will start to respond to infections present in the body. We hypothesize that, when food intake is improved after a long period of food shortage and nutrient deficiencies, the body may start to respond to *M*. *leprae*, causing the development of clinical disease.

This study has some limitations. First, data were collected referring to the period after the diagnosis of leprosy, which makes it hard to determine a causal relationship. However, the interviews were held shortly after diagnosis (maximum of nine months) and we specifically asked for changes in the patients’ diet and income after diagnosis, to be able to correct for this difference. Second, a cross-sectional design was employed because we aimed to collect data during a food shortage period. We assumed that the persons experiencing food shortage this year have also experienced this in the previous years and that their diets did not change over time. Ideally, a longitudinal study, collecting detailed data on diet and health, and taking blood samples to determine nutrient absorption, should be carried out to compare long-term data of the persons who developed leprosy with data of persons who did not. However, this will be a lengthy and expensive process. A third limitation is that most of the data were self-reported through questionnaires, introducing recall and response biases. Especially for very poor people with an unstable income their average income is difficult to estimate. By asking the same questions to cases and controls we tried to limit the effect on our results. In addition to the 24-hour recall, biomarkers for micro- and macronutrients in blood, urine and/or feces can be analyzed to assess dietary intake more objectively [41]. However, we decided not to use this method because collection and analyses of biomarkers is laborious and costly, especially because a high number of nutrients need to be analyzed since the key nutrients are unknown.

Fourth, the DDS might have been over or underestimated as it was based on one 24h-recall, which may not be representative of the usual diet. We tried to get the most reliable information possible by avoiding a recall for atypical days such as religious holidays. Furthermore, in developing countries diets tend to have a low day-to-day variability, thus one 24h-recall may be enough to get a good idea of the usual diet. In addition, when comparing population groups, a single dietary recall should give an accurate estimation of the intake of a whole group [42]. The DDS informs about the last 24 hours, while we extrapolated this to the diet before leprosy was diagnosed. Only few patients indicated that their food intake was different from that of last year, before diagnosis, however. Data of these patients were kept in the analyses, because there was little difference in the numbers of people who consumed more (4 patients) and who consumed less (5 patients) than in the period before they were diagnosed.

In conclusion, this study adds to the current knowledge on food shortage, nutrition and leprosy. We found that DDS and household food stocks are the most important diet-related factors associated with leprosy in Bangladesh. Furthermore, BMI and food expenditure per capita have a strong association with leprosy in our study. People living in poverty have less money available to spend on food. This results in a low consumption of animal source foods, fruits and vegetables. Deficiencies of the nutrients that these types of foods provide could result in an impaired immune response, which may be an explanation for the development of clinical leprosy. It is evident that little research has been carried out on the association between leprosy and nutrition, and that the immunological pathway leading to the clinical development of leprosy and the influence of nutrition should be studied further. Our results can be a starting point to elucidate the relation between nutrition and leprosy.

## Supporting Information

S1 Checklist STROBE checklist for case-control studies. (PDF)

## References

[1] Fine PE, Sterne JA, Ponnighaus JM, Bliss L, Saui J, et al (1997) Household and dwelling contact as risk factors for leprosy in northern Malawi. Am J Epidemiol 146: 91–102. 9215227 10.1093/oxfordjournals.aje.a009195

[2] van Beers SM, Hatta M, Klatser PR (1999) Patient contact is the major determinant in incident leprosy: implications for future control. Int J Lepr Other Mycobact Dis 67: 119–128. 10472363

[3] Moet FJ, Pahan D, Schuring RP, Oskam L, Richardus JH (2006) Physical distance, genetic relationship, age, and leprosy classification are independent risk factors for leprosy in contacts of patients with leprosy. J Infect Dis 193: 346–353. Epub 2005 Dec 2028. 16388481 10.1086/499278

[4] Deps PD, Guedes BV, Bucker Filho J, Andreatta MK, Marcari RS, et al (2006) Characteristics of known leprosy contact in a high endemic area in Brazil. Lepr Rev 77: 34–40. 16715688

[5] Richardus JH, Meima A, van Marrewijk CJ, Croft RP, Smith TC (2005) Close contacts with leprosy in newly diagnosed leprosy patients in a high and low endemic area: comparison between Bangladesh and Thailand. Int J Lepr Other Mycobact Dis 73: 249–257. 16830634

[6] World Health Organization (2013) Global leprosy situation: update on the 2012 situation. Weekly Epidemiological Record 88: 365–380. 24040691

[7] Kerr-Pontes LR, Barreto ML, Evangelista CM, Rodrigues LC, Heukelbach J, et al (2006) Socioeconomic, environmental, and behavioural risk factors for leprosy in North-east Brazil: results of a case-control study. Int J Epidemiol 35: 994–1000. Epub 2006 Apr 1027. 16645029 10.1093/ije/dyl072

[8] Feenstra SG, Nahar Q, Pahan D, Oskam L, Richardus JH (2011) Recent food shortage is associated with leprosy disease in Bangladesh: a case-control study. PLoS neglected tropical diseases 5: e1029 10.1371/journal.pntd.0001029 21572979 PMC3091833

[9] Abdullah M, Wheeler EF (1985) Seasonal variations, and the intra-household distribution of food in a Banlgadeshi village. Am J Clin Nutr 41: 1305–1313. 4003335 10.1093/ajcn/41.6.1305

[10] Hassan N, Huda SN, Ahmed A (1985) Seasonal patterns of food intake in rural Bangladesh. Ecology of Food and Nutrition 17: 175–186.

[11] Hillbruner C, Egan R (2008) Seasonality, household food security, and nutritional status in Dinajpur, Bangladesh. Food and Nutrition Bulletin 29: 221–231. 18947035 10.1177/156482650802900308

[12] Tetens I, Hels O, Khan NI, Thilsted SH, Hassan N (2003) Rice-based diets in rural Bangladesh: how do different age and sex groups adapt to seasonal changes in energy intake? Am J Clin Nutr 78: 406–413. 12936922 10.1093/ajcn/78.3.406

[13] Chandra RK (1997) Nutrition and the immune system: an introduction. Am J Clin Nutr 66: 460S–463S. 9250133 10.1093/ajcn/66.2.460S

[14] Wintergerst ES, Maggini S, Hornig DH (2007) Contribution of selected vitamins and trace elements to immune function. Ann Nutr Metab 51: 301–323. Epub 2007 Aug 2028. 10.1159/000107673 17726308

[15] Bangladesh Bureau of Statistics (2010) Report of the household income and expenditure survey 2010.

[16] Moet FJ, Pahan D, Oskam L, Richardus JH (2008) Effectiveness of single dose rifampicin in preventing leprosy in close contacts of patients with newly diagnosed leprosy: cluster randomised controlled trial. BMJ 336: 761–764. 10.1136/bmj.39500.885752.BE 18332051 PMC2287265

[17] Moet FJ, Schuring RP, Pahan D, Oskam L, Richardus JH (2008) The prevalence of previously undiagnosed leprosy in the general population of northwest Bangladesh. PLoS neglected tropical diseases 2. 10.1371/journal.pntd.0000198 PMC2254205 18301731

[18] Coates J, Swindale A, Bilinsky P (2007) Household Food Insecurity Access Scale (HFIAS) for Measurement of Food Access: Indicator Guide v. 3. Washington DC: Food and Nutrition Technical Assistance Project.

[19] Kennedy G, Ballard T, Dop M (2013) Guidelines for measuringhousehold and individual dietary diversity. Rome: FAO.

[20] Arimond M, Wiesmann D, Becquey E, Carriquiry A, Daniels MC, et al (2010) Simple food group diversity indicators predict micronutrient adequacy of women's diets in 5 diverse, resource-poor settings1–7. Journal of Nutrition 140: 2059S–2069S. 10.3945/jn.110.123414 20881077 PMC2955880

[21] Ruel MT (2002) Is dietary diversity an indicator of food security or dietary quality? A review of measurement issues and research needs, FCND Discussion Paper No.104. Washington DC: International Food Policy Research Institute 10.1177/156482650302400210 12891828

[22] Arimond M, Torheim LE, Wiesmann D, Joseph M, Carriquiry A (2009) Dietary Diversity as a Measure of the Micronutrient Adequacy of Women’s Diets: Results from Rural Bangladesh Site. Washington DC: Food and Nutrition Technical Assistance II Project, FHI 360 10.3945/jn.110.123612

[23] World Food Programme (2013) Bangladesh Food Security Monitoring Quarterly Bulletin.

[24] Thorne-Lyman AL, Valpiani N, Sun K, Semba RD, Klotz CL, et al (2010) Household dietary diversity and food expenditures are closely linked in rural Bangladesh, increasing the risk of malnutrition due to the financial crisis. Journal of Nutrition 140: 182S–188S. 10.3945/jn.109.110809 19923385

[25] Torlesse H, Kiess L, Bloem MW (2003) Association of household rice expenditure with child nutritional status indicates a role for macroeconomic food policy in combating malnutrition. J Nutr 133: 1320–1325. 12730417 10.1093/jn/133.5.1320

[26] Arsenault JE, Yakes EA, Islam MM, Hossain MB, Ahmed T, et al (2013) Very low adequacy of micronutrient intakes by young children and women in rural bangladesh is primarily explained by low food intake and limited diversity. Journal of Nutrition 143: 197–203. 10.3945/jn.112.169524 23256144

[27] Lockwood DNJ (2004) Commentary: Leprosy and poverty. International Journal of Epidemiology 33: 269–270. 15082625 10.1093/ije/dyh115

[28] Rashid DA, Smith L, Rahman T (2006) Determinants of dietary quality: evidence from Bangladesh. American Economics Association Annual Meeting. Long Beach, California.

[29] Bakker MI, Hatta M, Kwenang A, Van Mosseveld P, Faber WR, et al (2006) Risk factors for developing leprosy—a population-based cohort study in Indonesia. Lepr Rev 77: 48–61. 16715690

[30] Keusch GT (2003) The history of nutrition: malnutrition, infection and immunity. J Nutr 133: 336S–340S. 12514322 10.1093/jn/133.1.336S

[31] Scrimshaw NS, SanGiovanni JP (1997) Synergism of nutrition, infection, and immunity: an overview. Am J Clin Nutr 66: 464S–477S. 9250134 10.1093/ajcn/66.2.464S

[32] Diffey B, Vaz M, Soares MJ, Jacob AJ, Piers LS (2000) The effect of leprosy-induced deformity on the nutritional status of index cases and their household members in rural South India: a socio-economic perspective. Eur J Clin Nutr 54: 643–649. 10951513 10.1038/sj.ejcn.1601068

[33] Khadapani T, Mishra BK (2010) Health problems and nutritional status of selected leprosy victims of Burla town, Orissa, India. Current Research Journal of Social Sciences 2: 350–357.

[34] Montenegro RMN, Zandonade E, Molina MCB, Diniz LM (2012) Reactional state and nutritional profile among leprosy patients in the primary health care system, greater Vitoria, Espirito Santo State, Brazil. Cadernos de Saude Publica 28: 31–38. 22267063 10.1590/s0102-311x2012000100004

[35] Oh SY, Paik HY, Ju D (1998) Dietary habits, food intake and functional outcomes in those with a history of Hansen's disease in Korea. International Journal of Leprosy 66: 34–42. 9614838

[36] Rao PSS, John AS (2012) Nutritional status of leprosy patients in India. Indian Journal of Leprosy 84: 17–22. 23077779

[37] Harboe M (1985) The immunology of leprosy In: Hasting RC, editor. Leprosy. New York: Longman Group Ltd pp. 53–87.

[38] Ferguson A, Griffin GE (2000) Nutrition and the immune system In: Garrow JS, James WPT, Ralph A, editors. Human Nutrition and Dietetics. London: Elsevier Science Ltd pp. 747–764.

[39] Hirsch HH, Kaufmann G, Sendi P, Battegay M (2004) Immune reconstitution in HIV-infected patients. Clinical Infectious Diseases 38: 1159–1166. 15095223 10.1086/383034

[40] Lawn SD, Bekker LG, Miller RF (2005) Immune reconstitution disease associated with mycobacterial infections in HIV-infected individuals receiving antiretrovirals. Lancet Infectious Diseases 5: 361–373. 15919622 10.1016/S1473-3099(05)70140-7

[41] Jenab M, Slimani N, Bictash M, Ferrari P, Bingham SA (2009) Biomarkers in nutritional epidemiology: Applications, needs and new horizons. Human Genetics 125: 507–525. 10.1007/s00439-009-0662-5 19357868

[42] Gibson RS, Ferguson EL (2008) An interactive 24-hour recall for assessing the adequacy of iron and zinc intakes in developing countries. Washington, DC: IFPRI and CIAT.

